# The safety of omitting prophylactic abdominal drainage after laparoscopic liver resection: Retrospective analysis of 100 consecutive cases

**DOI:** 10.1016/j.amsu.2020.03.003

**Published:** 2020-04-02

**Authors:** Masaki Wakasugi, Junzo Shimizu, Yusuke Makutani, Chikato Koga, Masahiro Murakami, Haruna Furukawa, Toshinori Sueda, Tae Matsumura, Hiromichi Miyagaki, Mitsuyoshi Tei, Ryohei Kawabata, Junichi Hasegawa

**Affiliations:** Department of Surgery, Osaka Rosai Hospital, Osaka, Japan

**Keywords:** Laparoscopic liver resection, Prophylactic abdominal drainage, Postoperative management

## Abstract

**Introduction:**

Whether prophylactic abdominal drainage after laparoscopic liver resection (LLR) is necessary remains unclear. This study aimed to evaluate the safety of omitting prophylactic abdominal drainage after LLR.

**Methods:**

A retrospective analysis of 100 consecutive patients who underwent LLR at Osaka Rosai Hospital from April 2011 to November 2018 was performed. During this period, prophylactic abdominal drainage was routinely omitted during LLR without biliary anastomosis. The primary endpoint was the frequency of additional abdominal drainage. The secondary endpoint was the rate of postoperative complications.

**Results:**

Ninety-six patients (96%) underwent partial resection or lateral segmentectomy, and 89 patients (89%) were Child-Pugh grade A. The median operative time was 102 (range, 31–274) minutes. The median blood loss was minimal (range, 0–280 ml), and blood transfusion was performed for one patient (1%). One case (1%) was converted to open surgery. Additional abdominal drainage was required for one patient (1%) with an intraabdominal abscess. Postoperative complications were seen in 5 patients (5%). High-grade complications (≥grade III according to the Clavien-Dindo classification) were seen in two patients (2%). There were no cases of reoperation or perioperative death. The median postoperative hospital stay was 8 (range, 4–65) days.

**Conclusions:**

Prophylactic abdominal drainage could be safely omitted for selected patients and operative procedures.

## Highlights

• Prophylactic abdominal drainage for laparoscopic liver resection could be safely omitted for selected patients and operative procedures.
• Laparoscopic liver resection without prophylactic abdominal drainage might shorten patients' postoperative hospital stay.

## 1 Introduction

Prophylactic abdominal drainage after liver resection has been used to detect postoperative bleeding and bile leakage and to prevent fluid collection. Though some retrospective cohort studies and randomized, controlled trials have suggested that prophylactic abdominal drainage after liver resection might increase the risk of postoperative complications such as wound infection, retrograde abdominal infection, and ascitic fluid leakage [[1], [2], [3], [4], [5], [6]], prophylactic abdominal drainage after liver resection is still performed routinely in many hospitals. The advantages of laparoscopic liver resection (LLR) have been recognized by hepatobiliary surgeons, and LLR is gradually replacing conventional open liver resection in some experienced institutes [7]. However, there have been few reports on the safety of omitting prophylactic abdominal drainage after LLR [8]. The aim of this study was to examine the safety of omitting prophylactic abdominal drainage after LLR.

## 2 Patients and methods

### 2.1 Clinical setting

Medical records from all consecutive patients who underwent LLR at Osaka Rosai Hospital, Osaka, Japan from April 2011 to November 2018 were retrieved from a prospective database for this retrospective study. The patients who had a previous history of laparotomy or liver surgery were included in this study. During this period, prophylactic abdominal drainage during LLR without biliary anastomosis was routinely omitted in our hospital. The primary endpoint was the frequency of additional abdominal drainage. The secondary endpoint was the rate of postoperative complications.

### 2.2 Surgical technique

The patients were placed in the left half-lateral decubitus position for lesions in the right hepatic lobe or the supine position with legs apart for lesions in the left lobe under general anesthesia. Typically, LLR is performed with a 4- or 5-trocar technique. Pneumoperitoneum was monitored and maintained at 8–10 mmHg during laparoscopic surgery. Intraoperative ultrasonography was routinely performed to detect lesions and their relationships with major vessels in the liver parenchyma. The Pringle maneuver was used for intermittent clamping with 15 minutes of clamping and 5 minutes of declamping when needed and feasible. Microtase® (Alfresa Pharma Corporation, Osaka, Japan) was used for pre-coagulation before liver parenchymal transection. A Cavitron Ultrasonic Surgical Aspirator (CUSA®, Integra Life Sciences Corporation, Plainsboro, NJ, USA) and monopolar soft-coagulation and laparoscopic coagulation shears were used for liver parenchymal transection as needed. Fibrin sealant and polyglycolic acid felt was used to reduce biliary leakage and hemorrhage at the liver cut surface [9]. A prophylactic abdominal drain was routinely omitted.The resected specimen was isolated within a specimen bag, and retrieved from the umbilical wound.

### 2.3 Data collection and statistical analysis

The patients' characteristics, perioperative data, and postoperative complication were recorded. Results are expressed as medians (range) or numbers (percentages). The Clavien-Dindo classification [10,11] was used to evaluate postoperative complications. Student's *t*-test, Fisher's exact probability test, and the Mann-Whitney *U* test were used for the analysis of parametric and non-parametric data, as appropriate. Differences at p < 0.05 were considered significant. All statistical analyses were performed with EZR (Saitama Medical Center, Jichi Medical University, Saitama, Japan), which is a graphical user interface for R (The Foundation for Statistical Computing). More precisely, it is a modified version of R commander, designed to add statistical functions frequently used in biostatistics [12].

## 3 Results

Table 1 presents the characteristics of the 100 consecutive patients undergoing LLR at Osaka Rosai Hospital from April 2011 to November 2018. Fifty-one patients (51%) had a history of previous abdominal surgery, including 9 (9%) cases of liver resection. The median indocyanine green retention rate at 15 minutes (ICG-R15) was 14% (range, 0–63%). LLR was performed for malignancy in 96 patients [primary liver malignancy, *n* = 67 (67%); secondary liver malignancy, *n* = 29 (29%) and for benign lesions in the remaining 4 (4%) patients (hemangioma, *n* = 2; regenerative nodule, *n* = 1; inflammatory change, *n* = 1). The patients included 89 (89%) and 11 (11%) patients with Child-Pugh grades A and B, respectively.

**Table 1:** Patient characteristics.

Variable	*n* = 100
Age, *y*	72 (33–87)
Male sex	58 (58)
BMI, *kg/m*^*2*^	22.7 (15.4–38.5)
ASA score ≥ 3	14 (14)
Previous history of	
Abdominal surgery	51 (51)
Liver resection	9 (9)
Primary/Metastasis/Others	67/29/4
ICG-R15, *%*	14 (0–63)
Child-Pugh grade (A/B)	89/11
Liver damage (A/B/unknown)	71/22/7

Data are expressed as medians (range) or numbers (%), unless otherwise specified.

BMI: body mass index.

ASA: American Society of Anesthesiologists.

ICG-R15: indocyanine green retention rate at 15 min.

Table 2 shows the perioperative data. The median operative time was 102 minutes (range, 31–274 minutes). The median blood loss was minimal (range, 0–280 ml), and blood transfusion was performed for one patient (1%). One case (1%) was converted to open surgery due to injury of the intrahepatic biliary duct. Operative procedures were partial resection, subsegmentectomy, segmentectomy, hemiliver resection, and trisectionectomy in 79 (79%), 1(1%), 19 (19%), 1(1%), and 0 patients, respectively. The 19 patients undergoing segmentectomy included 17 cases of lateral segmentectomy. The median postoperative hospital stay was 8 days (range, 4–65 days).

**Table 2:** Perioperative data.

Variable	*n* = 100
Operative time, *min*	102 (31–274)
Blood loss, *ml*	minimal (0–280)
Blood transfusion	1 (1)
Conversion	1 (1)
Operative procedure	
Partial resection	79 (79)
lateral/S4/S5/S6/S7/S8/unknown	38/9/9/14/6/3
Subsegmentectomy	1 (1)
Segmentectomy	19 (19)
Hemihepatectomy	1 (1)
Trisectionectomy	0
Tumor size, mm	23 (10–75)
Resected liver weight, *g*	34 (4–342)
Prophylactic abdominal drainage	0
Postoperative hospital stay, *days*	8 (4–65)

Data are expressed as medians (range) or numbers, unless otherwise specified.

Table 3 shows the postoperative complications. Complications were seen in 5 patients (5%). No bile leaks were seen in this study. Additional abdominal drainage was required for one patient (1%) with an intraabdominal abscess. High-grade complications (≥grade III) were seen in two patients (2%). One patient (1%) with sepsis due to urinary tract infection was admitted to the intensive care unit. There were no cases of reoperation and no perioperative deaths in the present study.

**Table 3:** Postoperative complication.

	Clavien-Dindo classification grade					Total
	II	IIIa	IIIb	IV	V	
Complications, total	3 (3)	0	1 (1)	1 (1)	0	5 (5)
Bile leak	0	0	0	0	0	0
Aspiration pneumonia	1 (1)	0	0	0	0	1 (1)
Wound hemorrhage	2 (2)	0	0	0	0	2 (2)
Intraabdominal abscess	0	0	1 (1)	0	0	1 (1)
Urinary tract infection	0	0	0	1 (1)	0	1 (1)

Data are expressed as numbers (%).

## 4 Discussion

Laparoscopic partial resection or lateral segmentectomy without prophylactic abdominal drainage could be safely performed for patients with good liver function. In the present study, additional abdominal drainage was required for only one patient (1%) with an intraabdominal abscess, fewer than the previous reports (1–6, 8, 12; Table 4). In addition, postoperative complications were seen in only 5% of the patients, with no reoperations and no postoperative deaths. A large proportion of the present study population underwent partial resection or lateral segmentectomy without biliary anastomosis, and 89% of the patients were Child-Pugh grade A, which may have led to the safety and good operative outcomes of LLR without prophylactic abdominal drainage.

**Table 4:** Summary of hepatectomy and prophylactic abdominal drainage.

Author [reference]	Year	n	Open or laparoscopic	Bile leak, *%*	Additional drainage, *%*	Reoperation, *%*	Mean postoperative hospital stay, *days*	Mortality, *%*
Belghiti [1]	1993	39	open	5	16	3	12	3
Fong [2]	1996	60	open	6	18	0	13	3
Burt [3]	2002	981	open	N.A.	11	N.A.	10	2
Liu [4]	2004	52	open	0	0	2	13	2
Fuster [5]	2004	20	open	0	10	5	14	0
Sun [6]	2006	60	open	0	2	0	9	2
Ishizawa [8]	2014	298	laparoscopic	2	5	2	7	1
Wada [12]	2017	167	open^a^	2	6	1	14	0
Wakasugi	2019	100	laparoscopic	0	1	0	9	0

N.A.: not applicable.

a Including 31 cases (19%) of laparoscopically-assisted hepatectomy.

LLR without prophylactic abdominal drainage might shorten the postoperative hospital stay. The mean postoperative hospital stay of the present study was shorter than in previous reports, though the selection of the patients and the operative procedure were different (Table 4). Wada et al. [13] reported that the postoperative hospital stay of patients without prophylactic abdominal drainage (14 days) was significantly shorter than of those with abdominal drainage (18 days). Recently, the concept of Enhanced Recovery After Surgery (ERAS) has become widespread for patients undergoing hepatobiliary surgery [14]. According to a meta-analysis of randomized, controlled trials on the ERAS program, each ERAS protocol recommended that liver resection could be performed without prophylactic abdominal drainage [15].

Selection of the patients and the operative procedure is important for omitting prophylactic abdominal drainage after LLR and remains to be clarified. With regard to selection of patients, Liu et al. [4] reported that patients with chronic liver disease had higher postoperative complication rates related to prophylactic abdominal drainage, which resulted in a significantly longer postoperative hospital stay. They concluded that routine prophylactic abdominal drainage is contraindicated in patients with chronic liver diseases undergoing liver resection. On the other hand, Fuster et al. [5] demonstrated that prophylactic abdominal drainage decreased ascites leakage and significantly shortened postoperative hospital stay in their randomized, controlled trial in cirrhotic patients, and intraabdominal closed drainage is recommended for cirrhotic patients with clinically relevant portal hypertension. With regard to the operative procedure, several previous reports excluded patients with a potential risk of bile leakage (existence of bile leakage at the completion of the operation or requirement for biliary suturing or anastomosis) [[1], [2], [3], [4],^6^,^8^] or patients undergoing major liver resection [5] (Table 4). Fuji et al. [16] recommended superficial lesions in the lateral segment or exophytic lesions as the prime indications for LLR for patients with Child-Pugh B.

The present study had several limitations. First, the present study was carried out at a single institution and was retrospective in nature. Second, the present study is limited by the fact that a large proportion of the study population underwent partial resection or lateral segmentectomy without biliary anastomosis and had good liver function. Despite these limitations, the present study offers hepatobiliary surgeons valuable information regarding the safety of LLR without prophylactic abdominal drainage. Further large-scale, randomized, controlled trials are needed to confirm the results.

## 5 Conclusions

Prophylactic abdominal drainage could be safely omitted for selected patients and operative procedures. LLR without prophylactic abdominal drainage might shorten patients’ postoperative hospital stay.

## Ethical approval

This study has been approved by the ethics committee of our institution (receipt number 31–33) within which the work was undertaken and that it conforms to the provisions of the Declaration of Helsinki in 1995 (as revised in Edinburgh 2000). This study has been reported in line with the PROCESS 2018 criteria [17]. Written informed consent was obtained from the patients for the information to be included in our manuscript.

## Sources of funding

No sources of funding.

## Author contribution

Study design: MW.

Data collection:MW, YM, CK, MM.

Data analysis/interpretation: MW, JS, YM, CK, MM.

Paper writing: MW, JS.

Data interpretation: All.

Review: JS, JH.

## Trial registry number

Researchregistry 5300.

## Guarantor

MW.

## Provenance and peer review

Not commissioned, externally peer reviewed.

## Declaration of competing interest

No conflict of interest to declared.
